# Implant-associated malignancies in the genitourinary system: a comprehensive review of evidence and gaps

**DOI:** 10.1007/s11255-025-04712-x

**Published:** 2025-08-10

**Authors:** Shannon Francis, Jagmeet Arora, Jamasb Sayadi, Cindy Vu, Nada Raafat Khattab, Harshini Malapati, Thomas Johnstone, Jeong Hyun Ha, Yeonji Jang, Ekene Enemchukwu, Gordon Lee

**Affiliations:** 1 Stanford University School of Medicine, Stanford, CA USA; 2 School of Medicine, University of California, Irvine, Irvine, CA USA; 3 Department of Neurosurgery, Stanford University, Stanford, CA USA; 4 Stony Book University Hospital, New York, NY USA; 5 Interdisciplinary Program of Medical Informatics, Seoul National University College of Medicine, Seoul, South Korea; 6 Plastic and Reconstructive Surgery, CHA Gangnam Medical Center, CHA University School of Medicine, Pocheon, South Korea; 7 Department of Ophthalmology, Uijeongbu Eulji Medical Center and Eulji University, Uijeongbu, South Korea; 8 Department of Urology, Stanford University, Stanford, CA USA; 9 Department of Plastic Surgery, School of Medicine, University of California, Irvine, Irvine, CA USA

**Keywords:** Implant-related malignancy, Indwelling catheter, Catheter-associated carcinoma, Urologic oncology, Squamous cell carcinoma, Transitional cell carcinoma, Chronic mucosal irritation, Spinal cord injury, Implant material reporting, Prosthesis-related neoplasia

## Abstract

**Background:**

The relationship between foreign material implantation and cancer development has been investigated since the 1940s, yet many questions remain regarding the mechanisms and risks associated with these interactions. This scoping review examines the potential oncogenic effects of foreign material implantation within the genitourinary system (GUS), focusing on neoplasms linked to chronic mucosal irritation from medical devices.

**Methods:**

A systematic literature search of PubMed and Embase screened 15,925 studies for malignancies linked to implants (final search July 2023). Inclusion criteria involved clinical studies, cohort studies, case control studies, case reports and case series of human subjects with a history of genitourinary foreign devices or prosthesis implantation and de novo malignancies at the site of implantation or metastases of any tumor that were found adjacent to or near the prosthesis. Meta-analyses, systematic reviews, practice guidelines, narrative reviews, and studies with non-human subjects or benign masses were excluded. There were no date or language restrictions.

**Results:**

Twenty-six case reports and series (46 cases) and 21 cohort studies were identified. GU implants identified included transurethral and suprapubic catheters, transvaginal mesh, midurethral slings, and ureteral stents.

The mean duration of implant exposure before malignancy diagnosis was 16.8 years. Chronic irritation from indwelling catheters was frequently linked to malignancies, including squamous cell carcinoma (54.5%) and transitional cell carcinoma (38.6%), with the bladder as the most common tumor site (68.2%). However, materials used in catheters were rarely documented, underscoring a critical gap in reporting. Smoking, a significant bladder cancer risk factor, was often undocumented, complicating risk attribution.

**Conclusion:**

Our findings underscore the need for robust data on implant material and smoking history to refine our understanding of carcinogenesis. Clinicians should maintain vigilance for malignancy in patients with prolonged implant exposure, particularly in high-risk populations like those with spinal cord injuries. This review highlights the importance of balancing implant benefits with risks and provides guidance for future research and clinical practice.

**Supplementary Information:**

The online version contains supplementary material available at 10.1007/s11255-025-04712-x.

## Introduction

The association between implantation of foreign materials in the human body and subsequent development of cancer has been a longstanding and complex topic of investigation. The phenomenon, first described in the 1940s and termed the Oppenheimer effect, refers to the experimental observation that solid implants may promote carcinogenesis in rats [1]. In 1948, Oppenheimer began implanting plastic films under the abdominal skin of rodents, consistently inducing the development of sarcomas. Regardless of the chemical composition of the plastics used, malignant tumors, predominantly fibrosarcomas, developed within 1–2 years of implantation [2]. These groundbreaking experiments underscored the potential oncogenic effects of foreign materials and sparked decades of research exploring their relevance in human disease.

Over the years, research has demonstrated that this association is not merely theoretical but also clinically significant in humans for specific types of implants. One well-documented example is the link between textured breast implants and the development of breast implant-associated anaplastic large cell lymphoma (BIA-ALCL), a rare but serious malignancy [3]. Similarly, a robust association has been identified between indwelling catheters and bladder cancer, likely mediated by chronic irritation, inflammation, and subsequent malignant transformation [4]. These findings underscore the complex interplay between foreign materials, the host immune response, and carcinogenesis, sparking ongoing investigations into other implantable devices and their potential oncogenic risks.

However, not all foreign materials pose a significant cancer risk, and misconceptions have arisen in this context. For instance, polypropylene mesh, widely used in the treatment of pelvic organ prolapse and stress urinary incontinence, has been erroneously implicated in carcinogenesis. Despite rigorous studies demonstrating no causal link, misinformation continues to propagate, creating undue concern among patients and clinicians alike [5, 6]. This highlights the critical importance of evidence-based discussions regarding the safety of medical implants, particularly as the use of these devices continues to grow in modern medicine.

This scoping review synthesizes the current body of evidence on the development of de novo malignancies following the implantation of foreign materials within the genitourinary system (GUS). To our knowledge, this is the first review to focus specifically on implant-associated malignancies in the GUS. By examining the existing literature, we aim to provide clarity on the true oncogenic potential of various implants, identify gaps in current research, and propose directions for future investigation. This comprehensive review will contribute to a more nuanced understanding of the relationship between foreign materials and cancer development, ultimately guiding clinical practice and patient counseling.

## Methods

This scoping review was conducted using Preferred Reporting Items for Systematic Reviews and Meta-Analyses (PRISMA) guidelines [7]. As a review of existing literature, the study did not require Institutional Review Board (IRB) approval. With the assistance of an experienced medical librarian (E.W.), a systematic search was iteratively developed with study team input. The search was used in PubMed and Embase and broadly identified a large body of biomedical literature that located articles in which both cancer and prostheses/implants/devices were subjects. The final search, completed July 2023, for PubMed and Embase can be viewed in Appendix 1 of this article. Inclusion and exclusion criteria were developed and tested and then used to select articles in two stages (Table 1).

**Table 1:** MeSH/Search terms, inclusion and exclusion criteria

Inclusion	Exclusion
Implantation or use of a foreign device or prosthesis	Non-human subjects
Human subjects	Articles reporting outcomes related to benign masses (e.g., cysts, neuromas, papillomas, fibromas, and lipomas)
Studies examining external prostheses, e.g., pessaries, dentures, and indwelling catheters	No language restrictions
De novo malignancies at site of implantation OR De novo malignancies that were systemic or distant from the prosthesis occurring after prosthesis implantation/use OR Metastases of any tumor that were found adjacent to or near a prosthesis	No date restrictions
Clinical studies, cohort studies, case–control studies, case reports, case series, and conference abstracts	Meta-analyses, systematic reviews, practice guidelines, book chapters, narrative reviews, news, editorials, letters, comments

**Fig. 1:**
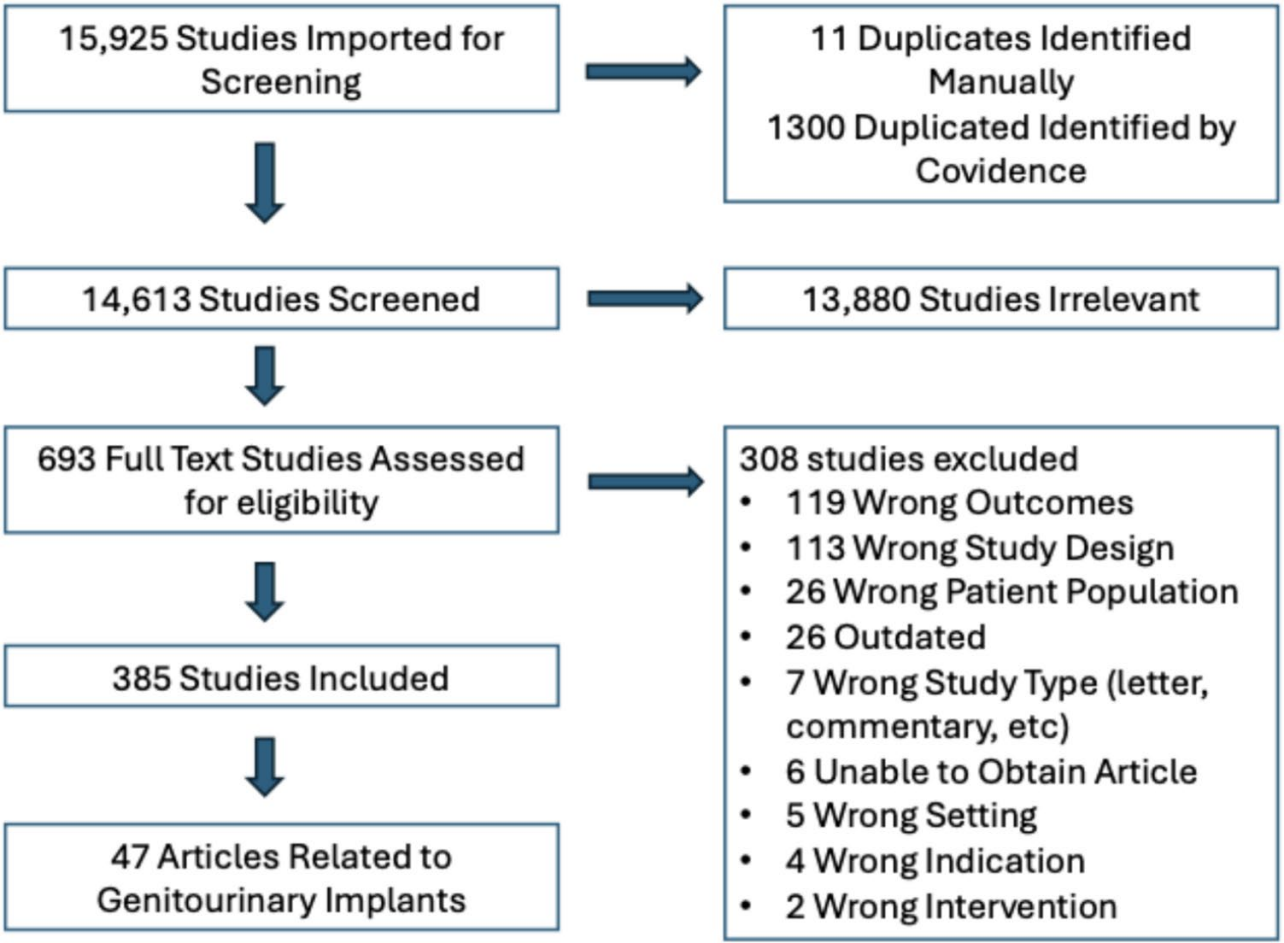
PRISMA Diagram

Covidence (Melbourne, Australia) was used for article screening. Titles and abstracts were reviewed initially. Full text of the articles retained after title and abstract screening were then reviewed. Articles were selected in duplicate and disagreements between reviewers were resolved by a third reviewer/subject expert. Cited reference searching of the final set of selected articles was carried out in Web of Science and Cochrane CENTRAL to identify papers not found in the search but which met the inclusion and exclusion criteria. See PRISMA flow diagram for details of selection (Fig. 1). The final set of articles was divided into section topics: genitourinary, craniofacial, orthopedics, vascular/cardiac, dental, neurology, breast, silicone non-breast implants, and miscellaneous. This review solely focuses on articles related to the genitourinary system.

Data was extracted using an extraction form developed and tested before use. The data fields for case reports included: study type, the language of the article, subject demographic information, subject comorbidities, subject history of prior malignancies, implant details (type, material, location), indications for implant, procedure performed for implant placement, malignancy details including histology, location, and stage, time from implant to presentation, presenting symptoms, and treatment details and course. Due to the heterogenous nature of the study populations and data collected in cohort studies, meta-analysis was not possible. The findings of the cohort studies were synthesized in the discussion.

## Results

Our scoping review identified 26 articles (case reports and case series), comprising of 46 reported cases, and 21 cohort studies. Of these, 12 case reports were published in foreign languages. Table 2 provides an overview of patient demographics and characteristics from the included case reports. The mean (SD) age of patients was 59.2 (± 13.0) years. Most patients were male (77.2%) and white (81.2%). Ten patients (22.7%) were non-smokers, while nine (20.4%) had a positive smoking history. Smoking history was not reported in the majority (56.8%) of case reports. The mean (SD) duration of implant exposure before presentation was 16.8 (14.9) years. Among patients, 20 (45.5%) had a suprapubic catheter, 17 (38.6%) had an indwelling urethral catheter, 2 (4.5%) practiced clean intermittent self-catheterization, 2 (4.5%) had ureterostomy tubes, 1 (2.3%) used ureteral stents, and 1 (2.3%) used urethral stents. One patient (2.3%) had a tension-free vaginal tape in the setting of clean intermittent catheterization. Additionally, three patients had a history of both suprapubic and indwelling urethral catheters. Other implants recorded included percutaneous nephrostomy tubes, intrapelvic foreign bodies from shrapnel wounds, and penile prostheses.

**Table 2:** Patient Demographics and Clinical Characteristics

*n*	44
Age (years) (mean [sd])	59.2 (13.0)
Sex (%)	
Male	34 (77.2)
Female	10 (22.7)
Race (5)	
White	36 (81.2)
Asian	7 (15.9)
Black	1 (2.2)
Smoking history (%)	
Not reported	25 (56.8)
Non-smoker	10 (22.7)
Smoker	9 (20.4)
Symptoms on presentation	
Hematuria	15 (34.1)
Chronic cystitis	12 (27.3)
Abdominal mass	9 (20.4)
Increased drainage around catheter site	8 (18.2)
Implant type	
Suprapubic catheter	20 (45.5)
Indwelling urethral catheter	17 (38.6)
Other	3 (6.8)
Clean intermittent self-catheterization	2 (4.5)
Ureterostomy tube	2 (4.5)
Ureteral stent	1 (2.3)
Urethral stent	1 (2.3)
Tension free vaginal tape	1 (2.3)
Indication for implant	
Spinal cord injury	30 (68.2)
Other	5 (11.4)
Neurogenic bladder	4 (9.1)
Strictures or obstruction of urinary tract	4 (9.1)
Vaginal wall repair	1 (2.3)
Implant material	
Not reported	41 (93.2)
Polyurethane	1 (2.3)
Polypropylene	1 (2.3)
Stainless steel alloy	1 (2.3)
Diagnosis	
Squamous cell carcinoma	24 (54.5)
Transitional (urothelial) carcinoma	17 (38.6)
Mucinous adenocarcinoma	2 (4.5)
Papillary carcinoma	2 (4.5)
Clear cell adenocarcinoma	1 (2.3)
Verrucous carcinoma	1 (2.3)
Tumor location	
Bladder	30 (68.2)
Suprapubic tract	6 (13.6)
Urethra	2 (4.5)
Other	3 (6.8)
Ureter	2 (4.5)
Anterior vaginal wall	1 (2.3)
Time exposed to implant (years) (mean [sd])	16.8 (14.9)

Appendix 2 details the implant type, indication, material, location, malignancy onset time, diagnosis, and treatment for the case reports, categorized by type of prosthesis and type of cancer (Appendix 2a and b, respectively). The majority of patients (68.2%) required implants due to spinal cord injuries. Other indications included neurogenic bladder (9.1%), strictures or obstructions of the urinary tract (9.1%), and less common causes such as anuria post-abdominal surgery, erectile dysfunction, urinary tract tuberculosis, and preparation for extracorporeal shock wave lithotripsy for staghorn calculi. The material composition of implants was not reported in most case reports (93.2%). Recorded materials included a polyurethane double J stent, a polypropylene transvaginal tape, and the stainless-steel alloy of the UroLume Stent. No case reports on indwelling catheters (suprapubic or transurethral) documented the type of material used. The most common presenting symptoms were hematuria (34.1%), chronic cystitis (27.3%), abdominal mass (20.4%), and increased drainage around the catheter site (18.2%). Two malignancies were diagnosed incidentally during cystoscopy. The majority of patients (54.5%) had squamous cell carcinoma (SCC), followed by transitional cell carcinoma (TCC) (38.6%). Less common histologies included mucinous adenocarcinoma (4.5%), papillary carcinoma (4.5%), clear cell adenocarcinoma (2.3%), and verrucous carcinoma (2.3%). Three patients presented with both SCC and TCC. The majority of tumors (68.2%) originated from the bladder. Additional locations included the suprapubic tract (13.6%), urethra (4.5%), ureter (4.5%), and anterior vaginal wall (2.3%). Rare sites included the junction of the renal pelvis, skin around a percutaneous nephrostomy site, and the glans penis with involvement of the urethral meatus.

## Discussion

Neoplasia is a well-documented response of the bladder epithelium to chronic irritation [8]. Neoplastic transformation of the urothelium typically progresses through stages: metaplasia (into squamous, columnar, or cuboidal epithelium), dysplasia, and ultimately transformation into transitional cell carcinoma (TCC) or squamous cell carcinoma (SCC). TCC may arise from the surface urothelium or from nonneoplastic proliferative lesions such as polypoid cystitis, in which villous projections of urothelium form in response to inflammation. SCC often arises from squamous metaplasia, a malignant precursor in which chronic irritation—due to infection, calculi, urinary retention, or long-term catheterization—replaces urothelium with stratified squamous epithelium [8].

Prolonged indwelling catheter use is a significant risk factor for such precursor lesions, including polypoid cystitis and squamous metaplasia, both of which may progress to malignancy. Among individuals with spinal cord injuries (SCI), who frequently rely on indwelling catheters, the prevalence of SCC is markedly higher than in the general population [9]. This increase is attributed to persistent epithelial irritation leading to metaplastic and neoplastic transformation. Our study is the first comprehensive review of genitourinary neoplasms associated with prosthetic materials.

### Catheters and SCC

The relationship between indwelling catheters and malignancy is most evident in SCI patients, who often rely on long-term catheterization. However, the etiology of bladder cancer in this population is debated. Some studies attribute the risk to catheter-induced chronic irritation, subclinical infections, or neurogenic bladder pathophysiology, independent of catheter use. Prolonged catheter exposure correlates with increased squamous metaplasia, and studies consistently demonstrate higher SCC rates in SCI patients using indwelling catheters compared to those using alternative bladder management methods [10–12]. One cohort study reported significantly higher bladder cancer risk in non-SCI patients with chronic indwelling catheter use, reinforcing catheter irritation as an independent carcinogenic factor. Indwelling catheters also increase susceptibility to recurrent urinary tract infections (UTIs), chronic cystitis, and subclinical infections, all of which contribute to persistent irritation. Retrospective analyses of SCI patients with bladder cancer revealed that all individuals with indwelling catheters were either colonized with bacteria or had recurrent UTIs [13, 14]. This highlights the role of infection-induced inflammation in bladder carcinogenesis. Emerging evidence suggests that the neurogenic bladder itself, regardless of catheter use, is a significant risk factor for bladder cancer. One study found that over 50% of SCI patients diagnosed with bladder cancer did not use indwelling catheters, suggesting that chronic inflammation from prolonged contact time between the bladder mucosa and stagnant urine in poorly emptying neurogenic bladders was a possible contributing factor [9]. A recent retrospective analysis found that only 4 out of 32 SCI patients who developed bladder cancer used indwelling catheters, suggesting that long-term indwelling catheter use is among multiple contributors to carcinogenesis in this population [15].

### Smoking and bladder carcinogenesis

Smoking is a well-established risk factor for bladder cancer, particularly TCC [16–18]. Meta-analyses have even shown that after 25 years of smoking cessation, the decrease in risk for bladder cancer still did not reach the level of non-smokers [19]. A recent Mendelian randomization study by Xiong [20] further solidifies this connection, demonstrating a causal relationship between smoking and increased bladder cancer risk [20]. However, many case reports and cohort studies fail to document patients’ smoking histories, limiting accurate risk attribution. Fifty-six percent of case reports did not report the smoking status of the patients, a critical oversight given the well-established relationship between smoking and bladder cancer. This omission is especially concerning in SCI populations, where smoking prevalence is higher than in the general population [21–24]. Without consistent reporting, it is difficult to separate the roles of catheter-induced irritation and smoking-related carcinogenesis.

For example, one retrospective analysis of SCI patients with bladder cancer found that the majority (81%) were diagnosed with TCC, contrasting with previous studies with a predominance of SCC [13]. Patients diagnosed with SCC also had a significantly longer time to diagnosis, compared to those with TCC. This may be due to one of two possibilities: (1) patients who developed SCC required a longer duration of catheter-induced irritation for squamous metaplasia to occur, or (2) SCC arose from a preexisting transitional cell tumor. TCC in these patients may have developed through a different mechanism, potentially influenced by lifestyle factors such as smoking or alcohol consumption. One retrospective cohort study of SCI patients with bladder cancer observed an incidence of SCC of 25%. Notably, the two patients who developed SCC did not have an indwelling catheter [25]. This may be due to 5 out of 8 patients having a smoking history, contributing to the development of TCC, rather than SCC. Accurate smoking data is crucial for delineating the relative contributions of lifestyle factors and catheter use to bladder cancer risk.

### Material contact and malignancy

The total contact of the implant with bladder tissue (both the amount of time and surface area) significantly impacts malignancy risk. Among our case reports, the average (SD) time exposed to an implant before presenting with malignancy was 16.8 (14.9) years. Indwelling catheter use exceeding 10 years is associated with a higher likelihood of bladder cancer [12, 26, 27]. Reducing contact time also delayed the onset of malignancy. Compared to patients who developed bladder malignancy after chronic indwelling catheter use, patients who used clean intermittent self-catheterization presented with malignancy on average 18 years later [28]. There is an interesting contrast in the reports of malignancies of the urinary tract with indwelling catheters versus ureteral or urethral stents. Our scoping review resulted in 37 case reports of malignancies resulting from indwelling catheters, but only 2 reports involving malignancies resulting from ureteral or urethral stents. This may be due to the reduced bladder surface area in contact with stents, compared to indwelling catheters, reducing the risk of chronic irritation and malignancy.

### Mesh and misconceptions

Direct contact between implants and mucosal tissue may influence carcinogenesis. Our scoping review identified four cohort studies on transvaginal mesh for pelvic organ prolapse or stress urinary incontinence. One matched cohort study found that transvaginal mesh was not associated with an increased risk of diagnosis of pelvic or local cancers at 1 year follow up, and during the entire follow-up period of 7 years [29]. The other three retrospective cohort studies on polypropylene midurethral slings showed no evidence to support an association between polypropylene mesh and the development of malignancy in humans [30–32]. The literature is consistent that there is no increased risk for pelvic cancers with transvaginal mesh placement. The reports of malignancy associated with transvaginal mesh contrasts with the heavily documented reports of bladder cancer in patients with indwelling catheters. This may be due to indwelling catheters directly contacting the mucosa, while transvaginal mesh is not. One case report of a bladder malignancy in a patient using clean intermittent catheterization had a tumor of squamous cell carcinoma directly arising from the dome of the bladder at the site of catheter contact [33]. Because the pathogenesis of malignant transformation involves squamous metaplasia, similar rates of implant associated malignancy with transvaginal mesh placement would not be expected. Additionally, only one case report involving penile prostheses and no reports involving artificial urinary sphincters, both of which are implants with no mucosal tissue contact, met our inclusion criteria for implant-associated malignancy.

### Underreported material composition

The material composition of implants is an important but often underreported factor. While polypropylene mesh used in transvaginal procedures is well-documented, catheter materials are rarely specified in studies. Only 6.8% of case reports identify the implant material, and no articles on indwelling catheters mention the type of material used. The most commonly used indwelling catheters in clinical practice are silicone-coated latex catheters, with pure silicone catheters serving as an alternative for patients with latex allergies [34]. While pure silicone catheters are more rigid and expensive, evidence suggests they reduce tissue inflammation and lower bacterial burden compared to silicone-coated latex catheters [35, 36]. A prospective study found that after 5 days of indwelling catheterization, pure silicone catheters exhibited significantly less bacterial colonization and biofilm formation than their silicone-coated latex counterparts [37]. Since subclinical infection and chronic inflammation are key drivers of neoplasia in long-term catheterization, the bacterial load accumulating over time may contribute to increased carcinogenic potential. Another material used for intermittent catheterization is styrene-butadiene rubber, commonly referred to as red rubber [38]. Widely used across various industries, red rubber has been extensively studied for its degradation into microplastics and their environmental and aquatic effects. However, its degradation producing cytotoxic free radicals requires extreme heat or UV exposure, making those results difficult to extrapolate to medical applications [39, 40]. Notably, the effects of styrene-butadiene rubber microplastics on the bladder epithelium remain largely unstudied. Given that patients may use catheters for years, it is critical to document catheter materials to isolate potential factors influencing carcinogenesis. With red rubber catheters causing varying levels of irritation or immune responses, consistent reporting of catheter materials is essential for assessing their role in bladder cancer risk.

Our study suggests that chronic mucosal irritation from urological implants, particularly indwelling catheters, may be a significant risk factor for bladder cancer. A limitation of this study is that included data are primarily from case reports and retrospective studies, limiting our ability to draw causal inferences. However, the lack of consistent reporting on smoking history and catheter materials limits our understanding of their interaction with bladder carcinogenesis. Future case reports and registries should standardize documentation of smoking history and implant material. Comprehensive data collection will clarify the mechanisms of implant-related carcinogenesis and develop strategies to reduce risk, particularly in high-risk populations such as SCI patients. Health care providers should be aware of the potential for malignancies to develop around prosthetic materials. This is particularly important when a patient presents with gross hematuria or an incidental bladder mass on imaging; neoplasia must be considered and assessed with appropriate imaging, cystoscopy and biopsy with careful histologic analysis.

## Supplementary Information

Below is the link to the electronic supplementary material.

Supplementary file1 (DOCX 16 KB)

Supplementary file2 (DOCX 57 KB)

## Data Availability

No datasets were generated or analysed during the current study.
