# Supplementary material for: Implant-associated malignancies in the genitourinary system: a comprehensive review of evidence and gaps
Source: Int Urol Nephrol. 2025 Aug 10;58(3):803–10. doi: 10.1007/s11255-025-04712-x (PMC12935859; doi:10.1007/s11255-025-04712-x)
Supplement: Supplementary file 1 — Supplementary file1 (DOCX 16 KB) [file 11255_2025_4712_MOESM1_ESM.docx]

**Appendix 1: Search Strategy**

**Pubmed search:**

(Neoplasms[majr] OR &quot;neoplasm&quot;[ti] OR &quot;neoplasm&quot;[ot] OR cancer[ti] OR

cancer[ot] OR tumor[ti] OR tumor[ot] OR tumour[ti] OR tumours[ot]) AND

(&quot;Prostheses and Implants&quot;[Majr] OR implant[ti] OR implant[ot]

&quot;Absorbable Implants&quot;[ti] OR &quot;Artificial Limbs&quot;[ti] OR &quot;Auditory Brain Stem

Implants&quot;[ti] OR &quot;Bioprosthesis&quot;[ti] OR &quot;Blood Vessel Prosthesis&quot;[ti] OR

&quot;Bone-Anchored Prosthesis&quot;[ti] OR &quot;Bone-Implant Interface&quot;[ti] OR &quot;Breast

Implants&quot;[ti] OR &quot;Cochlear Implants&quot;[ti] OR &quot;Dental Prosthesis&quot;[ti] OR

&quot;Crowns &quot; OR &quot;Dental Abutments&quot;[ti] OR &quot;Dental Clasps&quot;[ti] OR &quot;Dental

Implants &quot; OR &quot;Implant-Supported Dental Prosthesis&quot; OR &quot;Permanent

Dental Restoration&quot; OR &quot;Temporary Dental Restoration&quot; OR &quot;Dental

Veneers&quot;[ti] OR &quot;Dentures &quot; OR &quot;Palatal Obturators&quot;[ti] OR &quot;Periodontal

Prosthesis&quot;[ti] OR &quot;Artificial Tooth&quot; OR &quot;Implanted Electrodes&quot; OR

&quot;Implantable Defibrillators&quot;[ti] OR &quot;Implantable Neurostimulators &quot; OR

&quot;Embolic Protection Devices&quot;[ti] OR &quot;Vena Cava Filters&quot;[ti] OR &quot;Artificial

Eye&quot; OR &quot;Fiducial Markers&quot;[ti] OR &quot;Glaucoma Drainage Implants&quot;[ti] OR

&quot;Molteno Implants&quot;[ti] OR &quot;Heart Valve Prosthesis&quot;[ti] OR &quot;Artificial Heart&quot;

OR &quot;Heart-Assist Devices&quot;[ti] OR &quot;Experimental Implants&quot; OR &quot;Internal

Fixators&quot;[ti] OR &quot;Bone Nails&quot;[ti] OR &quot;Bone Plates&quot;[ti] OR &quot;Bone Screws &quot;

OR &quot;Bone Wires&quot;[ti] OR &quot;Suture Anchors&quot;[ti] OR &quot;Joint Prosthesis&quot;[ti] OR

&quot;Elbow Prosthesis&quot;[ti] OR &quot;Hip Prosthesis&quot;[ti] OR &quot;Knee Prosthesis&quot;[ti] OR

&quot;Metal-on-Metal Joint Prostheses&quot;[ti] OR &quot;Shoulder Prosthesis&quot;[ti] OR

&quot;Artificial Larynx&quot; OR &quot;Intraocular Lenses&quot; OR &quot;Multifocal Intraocular

Lenses&quot;[ti] OR &quot;Maxillofacial Prosthesis&quot;[ti] OR &quot;Mandibular Prosthesis&quot;[ti]

OR &quot;Orbital Implants&quot;[ti] OR &quot;Ossicular Prosthesis&quot;[ti] OR &quot;Penile

Prosthesis&quot;[ti] OR &quot;Prosthesis Design&quot; OR &quot;Prosthesis Coloring&quot; OR

&quot;Punctal Plugs&quot;[ti] OR &quot;Septal Occluder Device&quot;[ti] OR &quot;Stents&quot;[ti] OR

&quot;Drug-Eluting Stents&quot;[ti] OR &quot;Self Expandable Metallic Stents&quot;[ti] OR

&quot;Suburethral Slings&quot;[ti] OR &quot;Tissue Expansion Devices&quot;[ti:~1] OR &quot;Tissue

Scaffolds&quot;[ti] OR &quot;Artificial Urinary Sphincter&quot; OR &quot;Visual Prosthesis&quot;[ti]

OR mediport OR portacath OR &quot;vascular access devices&quot; OR &quot;indwelling

catheter&quot;[tiab:~1] OR Anophthalmia[ti] OR “Anophthalmic”[ti] OR

Anophthalmos[mh] OR “Orbital implants”[majr] OR “Orbital implants”[ti]

OR “Eye enucleation”[majr] OR “Eye evisceration”[majr] OR “Eye

evisceration”[tiab:~2] OR “Exenteration Eye”[tiab:~5])

**EMBASE search:**

'prostheses and orthoses'/exp/mj AND 'neoplasm'/exp/mj
