# Supplementary material for: Implant-associated malignancies in the genitourinary system: a comprehensive review of evidence and gaps
Source: Int Urol Nephrol. 2025 Aug 10;58(3):803–10. doi: 10.1007/s11255-025-04712-x (PMC12935859; doi:10.1007/s11255-025-04712-x)
Supplement: Supplementary file 2 — Supplementary file2 (DOCX 57 KB) [file 11255_2025_4712_MOESM2_ESM.docx]

Appendix 2a. Cases from literature, categorized by type of prosthesis

| Implant | Implant material | Implant location | Implant Indication | Age/Sex  race | Onset time (year) | Symptom | Diagnosis | Tumor location | Reference article |
| --- | --- | --- | --- | --- | --- | --- | --- | --- | --- |
| Central Venous Catheter | Not Reported | Superior vena cava | IgG infusions s/p bone marrow transplant | 51/F | 23 | Symptoms of Superior Vena Cava Syndrome | Malignant Fibrous Histiocytoma | Superior Vena Cava, originating from the tip of catheter | Churilla 2011 |
| Clean Intermittent Self-Catheterization | Not Recorded | Urethra | Spinal Cord Injury | 69/M, Asian | 2 | High grade fever | Transitional (Urothelial) Cell Carcinoma | Bladder | Sugimoto 1997 |
| Clean Intermittent Self-Catheterization | Not Recorded | Urethra | Underactive Bladder | 69/F | 4 | Intermittent hematuria for 4 months, pain on catheterization | SCC | Dome of Bladder Where the Catheter Impinged | Sene 1990 |
| Double J stent | Polyurethane | Ureter | Extracorporeal shock wave lithotripsy (ESWL) for staghorn caliculi | 33/M | 11 months | Tumor found upon planned J stent removal | Papillary Transitional Cell Tumor | Ureteral Orifice, Encasing the stent | Gron 1993 |
| Indwelling catheter | Not Reported | Urethra | Spinal Cord Injury | 69/M | 40 | Hematuria | SCC | Bladder | Parra 2007 |
| Indwelling catheter | Not Recorded | Urethra | Familial spastic paraplegia | 70/M | 50 | Hematuria | Urothelial Carcinoma | Bladder | Parra 2007 |
| Indwelling catheter | Not Reported | Urethra | Spinal Cord Injury | 72/M | 15 | Incidental finding during urodynamic study | Urothelial Carcinoma | Bladder | Parra 2007 |
| Indwelling catheter | Not Reported | Urethra | Multiple Sclerosis | 65/M | 22 | Urinary Urgency | Urothelial Carcinoma | Bladder | Parra 2007 |
| Indwelling Urethral Catheter | Not Recorded | Urethra | T9 Paraplegic | 46/M, White | 22 | Gross Hematuria | SCC | Right Lateral Aspect of Bladder | Locke 1985 |
| Indwelling Urethral Catheter | Not Recorded | Urethra | Spinal Cord Injury | 57/F, Asian | 20 | Macroscopic hematuria | SCC | Bladder | Sugimoto 1997 |
| Indwelling Urethral Catheter | Not Recorded | Urethra | T10 Paraplegic | 61/M | 2 | Not reported | Transitional (Urothelial) Cell Carcinoma | Bladder | Böthig 2017 |
| Indwelling Urethral Catheter | Not Recorded | Urethra | C5 Paraplegic | 51/M | 2 months | Not reported | SCC | Bladder | Böthig 2017 |
| Indwelling Urethral Catheter | Not Recorded | Urethra | L1 Paraplegic | 67/M | 21 months | Not reported | Transitional (Urothelial) Cell Carcinoma | Bladder | Böthig 2017 |
| Indwelling Urethral Catheter | Not Recorded | Urethra | Spinal Cord Injury | 38/F | 7 years | Hematuria and Chronic Cystitis | Transitional (Urothelial) Cell Carcinoma and SCC | Bladder | Melzak 1966 |
| Indwelling Urethral Catheter | Not Recorded | Urethra | Spinal Cord Injury | 39/M | Not specified | Chronic Cystitis and hematuria | SCC | Bladder | Melzak 1966 |
| Indwelling Urethral Catheter | Not Recorded | Urethra | Spinal Cord Injury | 58/M | 2 | Chronic cystitis and hematuria | Transitional Cell Carcinoma | Bladder | Melzak 1966 |
| Indwelling Urethral Catheter | Not Reported | Urethra | Spinal Cord Injury | 49/FM | 27 | Chronic UTI’s | SCC | Urethra | Dolinak 2007 |
| Intrapelvic foreign bodies due to shrapnel wounds | Not Recorded | Pelvis | Shrapnel Wounds | 63/M | 43 | Painless Hematuria and anemia | SCC | Left side of the bladder | Wyman 1988 |
| Midurethral tape - tension free vagina tape (TVT) | Polypropylene | Extrusion through transverse vaginal sulcus | Anterior and posterior vaginal wall repair | 58/F, Asian | 10 | UTI, acute retention | High grade Clear Cell Adenocarcinoma | Anterior vaginal wall; periurethral region next to synthetic tape | Lin 2016 |
| Penile prosthesis | Not Reported | Corpora Cavernosa | Erectile Dysfunction | 60/M, Asian | 7 | Mass on glans penis near urethral opening | Verrucous Carcinoma | Glans penis with involvement of the urethral meatus | Kim 2003 |
| Percutaneous nephrostomy (PCN) tube | Not Recorded | Lower back | Pelvi-ureteric Junction Obstruction | 38/F | 3 | PCN site skin growth and occasional bleeding | SCC | Skin around PCN site | Sharma 2019 |
| Suprapubic Catheter | Not Recorded | Bladder | T5 Paraplegic | 47/M | 12 | Non healing lesion within the suprapubic tract and microscopic hematuria | SCC | Dome of bladder | Locke 1985 |
| Suprapubic Catheter | Not Recorded | Bladder | L1 Paraplegic | 68/M | 10 | Not reported | Transitional (Urothelial) Cell Carcinoma | Bladder | Böthig 2017 |
| Suprapubic Catheter | Not Recorded | Bladder | Urinary Diversion for Urethral Strictures | 80/M, Black | 5 | Fungating Mass | SCC | Suprapubic tract, extending to the dome of the bladder | Stroumbakis 1993 |
| Suprapubic Catheter | Not Recorded | Bladder | Spinal Cord Injury | 71/M | 51 | Hematuria and Discharge from suprapubic cystostomy tube | Mucinous Adenocarcinoma | Anterior bladder extending along the suprapubic tube tract | Bauman 2015 |
| Suprapubic Catheter | Not Recorded | Bladder | T10 Paraplegic | 68/M | 39 | Presented with bloody drainage from suprapubic site | Mucinous adenocarcinoma | Suprapubic Cystostomy Tract | King 1997 |
| Suprapubic Catheter | Not recorded | Bladder | Paraplegia secondary to spinal tuberculosis | 55/M | 39 | Expanding, fungating lesion encircling the suprapubic catheter site | SCC | Suprapubic Cystostomy Tract | Massaro 2014 |
| Suprapubic Catheter | Not Recorded | Bladder | Idiopathic Urinary Retention | 85/F | 9 months | Increased discharge around surrounding catheter site | SCC | Suprapubic Cystostomy Tract, with expansion into bladder dome | Massaro 2014 |
| Suprapubic Catheter | Not Recorded | Bladder | Recurrent urethral strictures | 88/M | 25 | Fungating mass at suprapubic cystostomy tube site | SCC | Around the cystostomy tract without involving the bladder | Subramaniam 2017 |
| Suprapubic Catheter | Not Recorded | Bladder | Spinal Cord Injury | 38/M, Asian | 15 | Purulent discharge from cystostomy site for 4 months | Urothelial Carcinoma | Bladder, progressing along the cystostomy | Kohno 2013 |
| Suprapubic Catheter | Not Recorded | Bladder | Spinal Cord Injury | 54/M | 2 months | Chronic Cystitis | Transitional (Urothelial) Cell Carcinoma and SCC | Bladder | Melzak 1966 |
| Suprapubic Catheter | Not Recorded | Bladder | Spinal Cord Injury | 56/M | 3 | Hematuria and Chronic Cystitis | SCC | Bladder | Melzak 1966 |
| Suprapubic Catheter | Not Recorded | Bladder | Spinal Cord Injury | 37/M | 3 | Hematuria | SCC and transitional cell carcinoma | within suprapubic scar | Melzak 1966 |
| Suprapubic Catheter | Not Recorded | Bladder | Spinal Cord Injury | 55/M | 20 | Hematuria and Chronic Cystitis | SCC | Bladder, extending along suprapubic tract | Melzak 1966 |
| Suprapubic Catheter | Not Recorded | Bladder | Spinal Cord Injury | 58/M | 9 | Chronic Cystitis | SCC | Bladder | Melzak 1966 |
| Suprapubic Catheter | Not Recorded | Bladder | Spinal Cord Injury | 58/M | 35 | Inflamed Abdominal Mass | SCC | Abdominal mass surrounding the suprapubic cystostomy tube | Ito 2011 |
| Suprapubic Catheter | Not Recorded | Bladder | Spinal Cord Injury | 63/M | 37 | Large Suprapubic Mass | SCC | Suprapubic cystostomy tract, no invasion into bladder | Schaafsma 1999 |
| Suprapubic Catheter | Not Recorded | Bladder | Spinal Cord Injury | 50/M | 28 | Edema and sanguinous drainage at suprapubic cystostomy tube site, fever, chills | SCC | Suprapubic cystostomy tract, extending into bladder | Stokes 1995 |
| Suprapubic Catheter and Indwelling urethral catheter | Not Recorded | Bladder and urethra | Spinal Cord Injury | 48/F | Suprapubic catheter - 19 Indwelling Catheter - 7 | Hematuria and chronic cystitis | Transitional Cell Carcinoma | Bladder and suprapubic fistula | Melzak 1966 |
| Suprapubic Catheter and Indwelling urethral catheter | Not Recorded | Bladder and urethra | Spinal Cord Injury | 53/M | Suprapubic catheter - 16 months Indwelling urethral Catheter - 5 | Chronic cystitis | Transitional Cell carcinoma | Bladder | Melzak 1966 |
| Suprapubic Catheter and Indwelling urethral catheter | Not Recorded | Bladder and urethra | Spinal Cord Injury | 68/M | Suprapubic catheter - 8  Indwelling urethral Catheter - 5 | Chronic cystitis | Papillary carcinoma | Bladder | Melzak 1966 |
| Transurethral catheter | Not Recorded | Urethra | Spinal Cord Injury | 64/M | 12 | Incontinence and suprapubic and perineal pain | Urothelial Carcinoma | Bladder | Eichhorn 1984 |
| Ureteral catheter | Not Recorded | Ureter | Ureterocutaneostomy for urinary tract tuberculosis | 62/F, Asian | 31 | Tenderness and induration at ureterostomy site | SCC | Ureter | Sekine 1990 |
| UroLume Stent | Not Recorded | Ureter | Anuria after abdominal surgery | 77/F, Asian | 19 | Low back pain, cloudy urine discharge | Transitional Cell Carcinoma | Junction of the renal pelvis and ureter | Yamaguchi 1986 |
| Ureterostomy | Not Recorded | Urethra | Recurrent urethral strictures | 74/M | 10 | Bloody urethral discharge, weakened stream, hesitancy, microhematuria | Urothelial Carcinoma | Urethra, within the Urolume Stent | Paddack 2009 |

SCC, squamous cell carcinoma

Appendix 2b. Cases from literatures, categorized by type of cancer

| **Diagnosis** | **Implant** | **Age/Sex**  **race** | **Onset time** | **Symptom** | **Tumor location**  **(initial presentation)** | **Treatment** | **Reference article** |
| --- | --- | --- | --- | --- | --- | --- | --- |
| **Squamous Cell Carcinoma** | | | |  |  |  |  |
| SCC | Not Recorded | 69/F NA | 4 years | Intermittent hematuria for 4 months, pain on catheterization | Dome of Bladder Where the Catheter Impinged | Cystectomy and Urinary Diversion | Sene 1990 |
| SCC | Not reported | 69M, NA | 40 years | hematuria | Bladder | Cystoprostatectomy + Bricker | Parra 2007 |
| SCC | Not Recorded | 46/M, White | 22 years | Gross Hematuria | Right Lateral Aspect of Bladder | 4000 rad preoperative radiotherapy, then radical cystectomy with ileal conduit diversion | Locke 1985 |
| SCC | Not Recorded | 57F, Asian | 20 years | Macroscopic hematuria | Bladder | Radiation therapy | Sugimoto 1997 |
| SCC | Not Recorded | 51/M NA | 2 months | Not reported | Bladder | palliative chemotherapy | Böthig 2017 |
| SCC | Not Recorded | 39M, NA | Not specified | Chronic Cystitis and hematuria | Bladder | cystectomy and urinary diversion | Melzak 1966 |
| SCC | Not reported | 49F, NA | 27 years | Chronic urinary tract infections | Urethra | Not reported | Dolinak 2007 |
| SCC | Not Recorded | 63M/NA | 43 years | Painless Hematuria and anemia | Left side of the bladder | Not reported | Wyman 1988 |
| SCC | Not Recorded | 38F, NA | 3 years | PCN site skin growth and occasional bleeding | skin around PCN site | Wide local excision with 2 cm margins and simultaneous ureterocalicostomy | Sharma 2019 |
| SCC | Not Recorded | 47/M NA | 12 years | Non healing lesion within the suprapubic tract and microscopic hematuria | Dome of bladder | NA | Locke 1985 |
| SCC | Not Recorded | 80/M Black | 5 years | Fungating Mass | Suprapubic tract, extending to the dome of the bladder | Preoperative radiation (2000 rad), wide excision of mass, and partial removal of the bladder dome | Stroumbakis 1993 |
| SCC | Not recorded | 55/M NA | 39 years | 15 month hx of an expanding, fungating lesion encircling the suprapubic catheter site | Suprapubic Cystostomy Tract | Tumor excision and partial cystectomy | Massaro 2014 |
| SCC | Not Recorded | 85F, NA | 9 months | Increased discharge around surrounding catheter site | Suprapubic Cystostomy Tract, with expansion into bladder dome | Tumor excision and partial cystectomy | Massaro 2014 |
| SCC | Not Recorded | 88M, NA | 25 years | fungating mass at suprapubic cystostomy tube site | Around the cystostomy tract without involving the bladder | Surgical resection | Subramaniam 1993 |
| SCC | Not Recorded | 56M, NA | 3 years | Hematuria and Chronic Cystitis | Bladder | Cystectomy and urinary diversion | Melzak 2014 |
| SCC | Not Recorded | 55M, NA | 20 years | Chronic cystitis and hematuria | Bladder, extending along suprapubic tract | Cystectomy and urinary diversion, deep x ray treatment, and colostomy | Melzak 2014 |
| SCC | Not Recorded | 58M, NA | 9 years | chronic cystitis | Bladder | Cystectomy and urinary diversion, deep x ray treatment | Melzak 2017 |
| SCC | Not recorded | 58M, NA | 35 years | Inflamed abdominal mass | Abdominal mass surrounding the suprapubic cystostomy tube | Palliative external radiation therapy | Ito 2011 |
| SCC | Not recorded | 63M, NA | 37 years | Large suprapubic mass | Suprapubic cystostomy tract, no invasion into bladder | Surgical Excision | Schaafsma 1999 |
| SCC | Not recorded | 50M, NA | 28 years | Edema and sanguinous drainage at suprapubic cystostomy tube site, fever, chills | Suprapubic cystostomy tract, extending into bladder | Radial cystectomy with abdominal wall resection with ileal conduit | Stokes 1995 |
| SCC | Not Recorded | 62F, Asian | 31 years | tenderness and induration at ureterostomy site | Ureter | Palliative right ureterectomy, peplomycin and UFT | Sekine 1990 |
| **Transitional Cell Carcinoma** | | |  |  |  |  |  |
| Squamous Cell Carcinoma and Transitional cell carcinoma | Not Recorded | 37M, NA | 3 years | hematuria | Within suprapubic scar | cystectomy and urinary diversion | Melzak, J |
| Transitional (Urothelial) Cell Carcinoma | Not Recorded | 68/M NA | 10 years | Not reported | Bladder | palliative transurethral bladder tunor resection | Böthig R. |
| Transitional (Urothelial) Cell Carcinoma | Not Recorded | 69M, Asian | 2 years | high grade fever | Bladder | bilateral cutaneous ureterostomy and radiation therapy | Sugimoto K |
| Transitional (Urothelial) Cell Carcinoma | Not Recorded | 61/M NA | 2 years | Not reported | Bladder | radical cystectomy, and ureterocutaneostomy | Böthig R. |
| Transitional (Urothelial) Cell Carcinoma | Not Recorded | 67/M NA | 21 months | Not reported | Bladder | ileum conduit | Böthig R. |
| Transitional (Urothelial) Cell Carcinoma and squamous cell carcinoma | Not Recorded | 38F, NA | 7 years | Hematuria and Chronic Cystitis | Bladder | palliative care | Melzak, J |
| Transitional (Urothelial) Cell Carcinoma and squamous cell carcinoma | Not Recorded | 54M, NA | 2 months | Chronic Cystitis | Bladder | palliative care | Melzak, J |
| Transitional Cell carcinoma | Not Recorded | 58M, NA | 2 years | Chronic cystitis and hematuria | Bladder | cystectomy and urinary diversion | Melzak, J |
| Transitional Cell Carcinoma | Not Recorded | 48F, NA | Suprapubic catheter-19 years, Indwelling Catheter- 7 years | hematuria and chronic cystitis | Bladder and suprapubic fistula | cystectomy and urinary diversion, deep x ray treatment | Melzak, J |
| Transitional Cell carcinoma | Not Recorded | 53M, NA | Suprapubic catheter-16 months, Indwelling urethral Catheter- 5 years | chronic cystitis | Bladder | cancer discovered at autopsy | Melzak, J |
| Transitional Cell Carcinoma | Not reported | 77F, Asian | 19 years | low back pain, cloudy urine discharge | Junction of the renal pelvis and ureter | nephrectomy | Yamaguchi K |
| **Urothelial Carcinoma** | |  |  |  |  |  |  |
| Urothelial Carcinoma | Not recorded | 70M, NA | 50 years | hematuria | Bladder | Cystoprostatectomy + Bricker | Parra 2007 |
| Urothelial Carcinoma | Not reported | 72M, NA | 15 years | Incidental finding during urodynamic study | Bladder | Transurethral resection (TUR) | Parra 2007 |
| Urothelial Carcinoma | Not reported | 62M, NA | 22 years | Urinary Urgency | Bladder | TUR + BCG | Parra 2007 |
| Urothelial Carcinoma | Not Recorded | 38M, Asian | 15 years | purulent discharge from cystostomy site for 4 months | Bladder, progressing along the cystostomy | four courses of gemcitabine-cisplatin (GC) chemotherapy, total cystectomy and ileal conduit surgery | Kohno 2013 |
| Urothelial Carcinoma | Not reported | 64M, NA | 12 years | incontinence and suprapubic and perineal pain | Bladder | cystectomy | Eichhorn 1984 |
| Urothelial Carcinoma | Stainless steel alloy | 74M, NA | 10 years | bloody urethral discharge, weakened stream, hesitancy, microhematuria | Urethra, within the Urolume Stent | Urethrectomy, radical perineal prostatectomy, and subtotal penectomy with negative margins; later he further underwent bilateral pelvic lymph node dissection with ileocystoplasty + catheterizable stoma | Paddack 2009 |
| **Other** |  |  |  |  |  |  |  |
| High grade Clear Cell Adenocarcinoma | Polypropylene | 58F, Asian | 10 years | UTI, acute retention | Anterior vaginal wall; periurethral region just next to synthetic tape | Partial transobturator tape excision, palliative radiation therapy | Lin 2016 |
| malignant fibrous histiocytoma | Not reported | 51F, NA | 23 years | Symptoms of Superior Vena Cava Syndrome | Superior Vena Cava, originating from the tip of catheter | Surgical excision | Churilla 2011 |
| Mucinous Adenocarcinoma | Not Recorded | 71/M NA | 51 years | Hematuria and Discharge from suprapubic cystostomy tube | anterior bladder extending along the suprapubic tube tract | cystoprostatectomy, abdominal wall resection, ileal conduit creation, and abdominal wall reconstruction. | Bauman 2015 |
| Mucinous adenocarcinoma | Not Recorded | 68/M NA | 39 years | Presented with bloody drainage from suprapubic site | Suprapubic Cystostomy Tract | Surgical resection | King 1997 |
| Papillary carcinoma | Not Recorded | 68M, NA | Suprapubic catheter-8 months, Indwelling urethral Catheter- 5 years | chronic cystitis | Bladder | cancer discovered at autopsy | Melzak 1966 |
| papillary transitional cell tumor | Polyurethane | 33M, NA | 11 months | Tumor found upon planned J stent removal | ureteral orifice, encasing the stent | transurethral resection and stent removal | Grøn 1993 |
| Verrucous Carcinoma | Not Reported | 60M, Asian | 7 years | Mass on glans penis near urethral opening | Gland penis with involvement of the urethral meatus | Total excision fo the glans penis | Kim 2003 |

SCC Squamous Cell Carcinoma
